# Assessing the Effects of Medical Information on Parental Self-Medication Behaviors for Children’s Health: A Comparative Analysis

**DOI:** 10.3390/medicina59122093

**Published:** 2023-11-29

**Authors:** Petruța Tarciuc, Alina Duduciuc, Sergiu Ioachim Chirila, Valeria Herdea, Oana Rosu, Andreea Varga, Ileana Ioniuc, Smaranda Diaconescu

**Affiliations:** 1 Doctoral School, “George Emil Palade” University of Medicine, Pharmacy, Science, and Technology of Târgu Mures, 38 Gheorghe Marinescu Str., 540139 Targu Mures, Romania; 2 Faculty of Communication and Public Relations, National University of Political Studies and Public Administration, 012104 Bucharest, Romania; 3 Medical Informatics and Biostatistics, Faculty of Medicine, Ovidius University, 900527 Constanta, Romania; 4 Department ME2-Clinical Disciplines, “George Emil Palade” University of Medicine, Pharmacy, Science, and Technology of Târgu Mures, 540139 Targu Mures, Romania; 5 Department of Internal Medicine II-Cardiology, Emergency Clinical County Hospital, 540042 Targu Mures, Romania; 6 Faculty of Medicine, “Grigore T. Popa” University of Medicine and Pharmacy, 16 Universitatii Str., 700115 Iasi, Romania; 7 Faculty of Medicine, “Titu Maiorescu” University of Medicine, 67A Gheorghe Petrascu Str., 031593 Bucharest, Romania

**Keywords:** self-medication, children, family doctors, Health Belief Model

## Abstract

*Background and Objectives*: Health professionals have voiced concerns about the danger of self-medication in times of growing use of over-the-counter medicines and, in some contexts, the unregulated selling of them. Previous research has examined the incidence of parental self-medication as well as the use and abuse of antibiotics without medical advice. However, these studies have limited evidence on the role of family doctors and the perceived severity of self-medication in the case of parents. Based on the Health Belief Model, our research tested the effects of exposure to medical information on the parents’ attitudes toward self-treating their children, without medical advice. Specifically, we aimed to assess whether exposure to information warning about the risks of treating children without a medical prescription influences parents’ attitudes toward administering medicines to their children without medical advice. *Materials and Methods*: 210 parents engaged in the study, and were divided into two groups. One group was exposed to educational materials related to the perils of self-medication and the second one was not. All participants answered the same questionnaire and the answers were compared between the two groups. *Results*: The results showed that our respondents evaluated the practices of self-medication negatively (a higher score indicates a more negative evaluation), especially when it came to treating their children without medical advice (3.91 ± 1.04 for unexposed and 3.98 ± 1.08 for exposed). However, their attitudes towards self-medication varied depending on their beliefs about administering certain medications. Both those exposed to the warning information and those who were not exposed have agreed that they are unable to avoid treatment of their ill child without medical advice. *Conclusions*: In general, our respondents evaluate negatively the practices of self-medication, especially the treatment of their children without medical advice. Therefore, future health education campaigns need to be targeted specifically, with messages that guide how to act in particular cases depending on the medication used and the child’s condition.

## 1. Introduction

Self-medication (SM) is a complex concept influenced by various defining factors. Authors often consider how individuals obtain medications, the absence of professional healthcare guidance, the source of medications, and the reasons behind self-medication [1].

The World Health Organization (WHO) defines self-medication as the use of medicinal products by individuals to address self-identified health issues or symptoms [2]. One important issue in this regard is the widespread phenomenon of parental self-medication, a practice observed globally. Research studies have reported varying prevalence rates, ranging from 25.2% to 96% in different countries [3]. The COVID-19 pandemic has further amplified the tendency toward self-medication, primarily due to the difficulties associated with accessing healthcare services [4,5].

In Romania, the number of doctors and nurses per capita is significantly lower than the EU average, primarily due to the emigration of medical personnel. In 2019, there were 3.2 practicing doctors per 1000 inhabitants, which was one of the lowest ratios in the EU (the EU average being 3.9), and 7.5 nurses per 1000 inhabitants (the EU average being 8.4). This negatively affects access to care and contributes to increased waiting times [6].

Parents often manage their children’s symptoms like fever, cough, cold, or diarrhea without prior consultation with a healthcare professional [4]. The utilization of analgesics, antipyretics, anti-inflammatory agents, and cough or cold remedies is a recurring pattern in pediatric self-medication, with paracetamol (acetaminophen) being one of the most frequently employed non-prescription analgesics and antipyretics [7,8].

Children are still in the developmental stage, with different pharmacokinetic and pharmacodynamics characteristics [9]. Parents resort to self-medication for their children’s fever without understanding the underlying cause or appropriate treatment [10,11]. They are prone to errors due to their limited understanding of medication, including dosage, timing, preparation, and drug interactions, as well as inadequate knowledge regarding the selection, acquisition, storage, and disposal of medicines [9,12,13]. Special attention should be paid to OTC medications with active substances that have age and weight-related contraindications. Additionally, scarce knowledge of the toxicity of medications generates a considerable underestimation of the risks [14].

The pharmacist is the one who intervenes in the education of people regarding the correct use of OTC medicines [15]. In a study conducted by Bedhomme, S. et al., in a community pharmacy, the pharmacist’s correction of doses and type of OTC medicine was accepted in 94.8% of parental requests for an OTC medicine in pediatric self-medication [16], and 73.3% of OTC-medicine-related problems were resolved by the pharmacist’s intervention alone, mostly by counseling and drug switching [17]. Research revealed a concerning trend as the majority of the surveyed pharmacists provided incorrect responses about the administration of the drug with food. This can lead to a loss of therapeutic effectiveness or drug toxicity. Is essential to update pharmacists’ knowledge of dispensed drugs and so to enhance patient safety and care [18].

While the usage of over-the-counter (OTC) medications for children raises concerns about their potential risks and unproven efficacy, studies have identified the motivators that lead parents to choose self-treatment of their children. These motivations often include factors like accessibility, convenience, and time-saving benefits [19]. In certain qualitative inquiries, parents have cited the use of OTC medicines as a means of gaining control over their children’s behavior, alleviating discomfort associated with a sick child [20]. Research indicates that self-medication behaviors are strongly influenced by non-cognitive factors, such as habits and symptom affinity, while cognitive factors like knowledge and risk comprehension exhibit limited association [21].

To gain deeper insights into the factors driving self-medication behaviors and to develop effective prevention strategies, various models have been employed. The Health Belief Model emerges as a valuable tool for examining the relationship between health beliefs and self-medication behaviors [22,23]. According to this model, individuals are more inclined to engage in health-promoting practices when they perceive a health risk because they believe they can mitigate it and are motivated to do so.

Several studies have employed the Health Belief Model to investigate self-medication among parents. Findings suggest that educational interventions based on this model can enhance parents’ perceived self-efficacy, reduce barriers to the timely addressing of their child’s health issues, and improve medication adherence [24].

Our research employs the Health Belief Model as a framework to understand how patients’ attitudes towards self-medication for children is influenced by exposure to information that emphasizes the risks associated with treating them without a medical prescription.

## 2. Materials and Methods

### 2.1. Methods, Sample, Research Instruments, and Data Analysis

To fulfill the objectives of this research, a survey based on a questionnaire was developed by a panel of specialists in medicine, psychology, and communication sciences. A total of 250 adult participants, each with at least one child, were invited to partake in this study. These individuals were selected randomly from the patient databases of two general practitioners situated in Bucharest, Romania. Among these invitees, 210 willingly consented to engage in the study, yielding a commendable response rate of 80%. Subsequently, these 210 participants were subject to random allocation into two distinct groups: one designated as Group 1 (G1 = 108), which received exposure to medical information concerning the perils of self-medication. This information was conveyed through the Romanian translation of educational materials akin to those disseminated by the United States Food and Drug Administration (FDA) addressing the use of over-the-counter (OTC) medications for children [25]. In contrast, the other group, denoted as Group 2 (G2 = 102), did not receive such medical information.

The distribution of the questionnaire, accompanied by data protection documentation and informed consent, was carried out via email. It was explicitly communicated to respondents that no personally identifiable information, including names, addresses, email addresses, or health statuses, would be collected as part of this research endeavor. Participation in the study was entirely voluntary, and respondents were afforded the choice to abstain from questionnaire completion if they wished not to participate or to proceed with survey submission.

The questionnaire itself comprised a total of 20 inquiries. To gauge the influence of exposure to medical information on self-medication practices, two distinct versions of the questionnaire were crafted. One version was intended for parents who had received pertinent medical information concerning the hazards associated with self-administering medication to their children. In this scenario, the medical information closely mirrored the FDA’s messages pertaining to the risks of parents medicating their children without prior medical consultation. The second version, devoid of any medical educational content, was designated for the remaining participants. Both versions, despite this difference, incorporated identical questions designed to evaluate the following aspects:

1. Attitude of parents towards self-medication of their children. To avoid latent respondents’ biases, we addressed an indirect question where the participants assessed (on a 5-point Likert scale, where 5 means strongly agree and 1 means strongly disagree) the case of a target mother who self-treated the fever of her two-year-old child without medical advice.
2. Perceived severity of self-medication, in this case, the severity of side-effects of self-medication. The second variable measured in the questionnaire was the perceived severity of self-medication, specifically the severity of side effects resulting from self-medication. Respondents were asked to indicate their level of agreement or disagreement (on a 5-point Likert scale) with the statement “If I give medicine to my child without medical advice, there may be other side consequences/complications for their health.”.
3. Perceived susceptibility to self-medication, which refers to the extent to which respondents perceive the risks of self-medicating their children with a specific medication, such as acetaminophen. To measure this variable, we included a statement (e.g., “Administering acetaminophen to my child without medical advice could be harmful”) to which respondents indicated their level of agreement or disagreement on a 5-point Likert scale.
4. Perceived barriers to adopting appropriate health behavior. Perceived barriers to seeking medical advice refer to the obstacles or challenges that might prevent parents from adopting appropriate health behavior. In this study, we assessed the perceived quality of the relationship between respondents and their family doctor (GP), using a 5-point Likert scale.
5. Perceived self-efficacy. Perceived self-efficacy refers to the extent to which respondents believed they could follow the advice of medical professionals and avoid self-medicating their children. This construct was measured through a statement (I can avoid treating my child’s illness without medical advice), to which participants expressed their level of agreement or disagreement using a 5-point Likert scale.
6. The beliefs regarding the use of over-the-counter medicine from the pharmacy. The questionnaire included a statement to measure the beliefs of respondents regarding the use of over-the-counter medicine from the pharmacy. The statement read, “If purchasing over-the-counter medication is legal, then a doctor’s advice is not necessary.” Respondents were asked to express their agreement or disagreement on a 5-point Likert scale.
7. Socio-demographics. Information about the respondents’ socio-demographic characteristics (e.g., age, gender, education, and number of children) was also collected during the study.

The data were collected between October and November 2022.

### 2.2. Statistical Analysis

To analyze the collected data, statistical analyses were conducted using SPSS 27 software. We used descriptive statistics (counts, percentages) to present the results or mean and standard deviation (SD). To investigate associations within the data, the Chi-square test was employed, while for comparing results, the two tailed independent t-test was applied, with effect sizes reported using Cohen’s d. The choice of the t-test was informed by the substantial sample sizes in each group, exceeding 100 participants, and was consistent with previous research findings that indicated negligible differences between parametric and nonparametric tests when dealing with Likert scales [26,27].

Each response on the 5-point Likert scale was assigned a numerical value, ranging from 1 for complete disagreement to a maximum of 5 for total agreement.

To ascertain the independent influence of various socio-demographic factors on respondents’ agreement with the survey statements, ordinal regression analyses were conducted. These analyses were executed in situations where statistically significant differences were discerned between the exposed and unexposed groups. In all such scenarios, none of the socio-demographic variables emerged as statistically significant contributors in modeling the responses.

Table 1 presents the socio-demographic characteristics of the sample. We identified that participants within the exposed group were younger when compared to the unexposed group. Another significant difference between the two groups was related to the percentage of participants within the rural areas, where a significantly higher percentage had residence in such environments. For all other characteristics, differences were not statistically significant.

## 3. Results

We investigated the relationship between the assessment of the GP’s activity and the assessment of the health system. These aspects might play an important role in the parents’ decision to self-treat their children. After being exposed to the medical information, we observed that there was no statistically significant difference between the two groups of parents (Table 2). Instead, we observe a very high level of trust in their personal GP for all participants, with an average, overall score of 4.23 and an SD of 1.12 (maximum score is 5). At the same time, we observed a significantly lower score for trust in the health system, with an overall average of 2.56 and a standard deviation (SD) of 1.03.

The way parents answered the questions evaluating their attitudes toward self-medication is depicted in detail in Supplementary Materials no. S1.

We evaluated the participants’ beliefs regarding the use of over-the-counter medicines in general and acetaminophen in particular.

**Figure 1:**
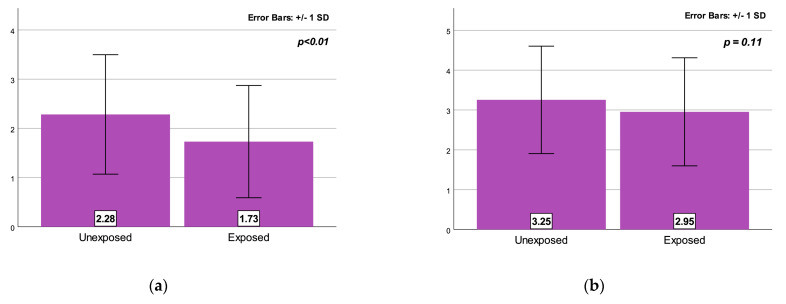
(**a**) Group difference (means) on the beliefs regarding the use of the over the counter medicine. (**b**) Group difference (means) on the beliefs regarding the use of acetaminophen when children are ill.

On average, respondents not exposed to medical information had an average score of 2.28, with an SD of 1.21. Almost one-quarter of the unexposed participants in the study declared to be neutral (23.5%). For the exposed group, the average score was 1.73, with an SD of 1.14, and 13% neutral (Figure 1a). The result of the t-test is statistically significant (t = 3.4, df = 208, *p* < 0.001), with a mean difference of 0.55 (95% CI 0.16–0.23). The effect size, calculated using Cohen’s d was medium, with a value of 0.47 (95% CI 0.19–0.74). Thus, participants unexposed to educational material tended to believe that medications obtained from a pharmacy without a medical prescription can be administered without medical advice, while participants within the exposed group tended not to embrace this idea. Therefore, a significant difference between the two groups was obtained showing that our H6 hypothesis was sustained.

Furthermore, respondents not exposed to medical information tended to agree that medications for children obtained from a pharmacy, without a medical prescription (i.e., acetaminophen), can be administered to children without the advice of a doctor (the average score is 3.25, with an SD of 1.35). On the other hand, respondents warned of the risks of self-medication tended not to embrace this idea, having a lower average score, of 2.95, with an SD of 1.36. No statistical difference between the two groups (t = 1.61, df = 208, *p* = 0.11) was obtained (Figure 1b).

Regarding the other variables measured according to the HBM model—perceived severity, perceived self-efficacy, and perceived barriers—all these were not statistically significant for self-medication cognitions. For example, both those exposed to the warning information and those who were not exposed agreed that they are unable to avoid treatment of their ill child without medical advice, although they disagreed with all self-medication practices (Table 3).

A statistically significant, positive, weak correlation (r = 0.258, *p* < 0.01) was obtained when we tested the relation between the level of satisfaction with the GP’s advice and the level of the perceived threat of self-medication (Table 4). This holds the second hypothesis (H2) of our study, which suggests that when the family doctor is highly regarded, the perception of the risk of self-treating the child is also high and vice-versa.

**Figure 2:**
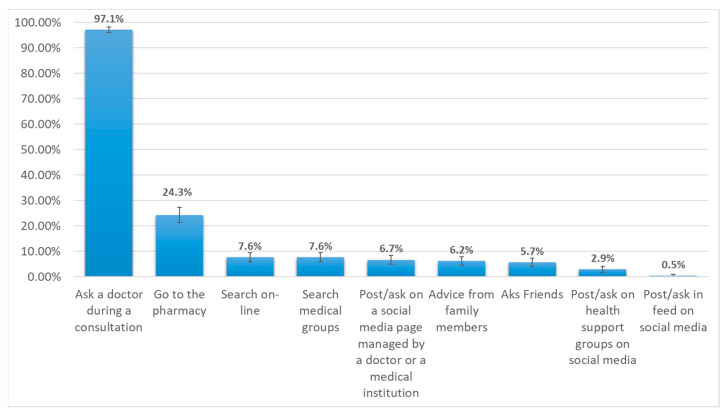
Methods used by the participants to reduce uncertainty. (Each bar represents the percentage from the total number of participants).

Another evaluated aspect was related to the methods used by the parents to seek advice related to the child’s current episode of disease. This was a multiple-response question and the answers of all the participants were analyzed for all 210 participants. The responses favor the idea of being consulted by a medical doctor, with almost all of the parents choosing this option. Another method of getting help was to consult directly with a pharmacist, which was a usual attitude for approximately one-quarter of the parents (24.3%). This indicates a high degree of trust in direct contact with health personnel, such as a doctor or a pharmacist. On the other hand, regarding methods that mainly rely on obtaining advice from friends or familiar people, each one is usually used by approximately 5% of the parents. Even though Internet resources and social media seem to play a very important role in adults’ lives, different methods of communicating with specialists in the field or getting help from peers from social media groups were generally low in our sample, with percentages ranging from 0.5% to 7.6% (Figure 2).

## 4. Discussion

Our findings have highlighted four important pieces of data. Firstly, parents’ beliefs about the use of over-the-counter medicines—specifically their beliefs about the purchase and use of OTCs when their child is sick—prove to be a significant variable in explaining the different effects of exposure to medical warning communication on self-medication risks. Secondly, there is a relationship between the level of satisfaction with the family doctor and the perceived threat of self-medication. This suggests that when a patient is satisfied with the general advice provided by their GP, he/she will be more aware of the risks of self-medicating without medical advice. Thirdly, both parents who were exposed to medical information and those who were not warned about the risks of self-medication reported feeling “helpless” to avoid self-medication when their child is sick, which in terms of the HBM model means a low level of perceived self-efficacy or those beliefs that avoiding self-medication is risky. Even if they have overall negative attitudes about self-medication and know that it should generally be avoided, they respond that they cannot avoid it in a specific context. Fourthly, the variables measured according to the HBM model—perceived severity, perceived self-efficacy, and perceived barriers—were not found to be statistically significant concerning self-medication cognitions.

Research suggests that perceived susceptibility to illness is an important predictor of preventive health behaviors [28]. Our study reports a statistically significant difference in perceived susceptibility after the educational intervention between the intervention and control group, which could be a meaningful indication of the effect of the educational intervention (information warnings in these cases) on improving the perceived susceptibility of individuals in the intervention group. In many other studies, it has been shown that if the intervention based on HBM affected and raised the susceptibility, then the probability of risky behaviors was decreased [23,29,30,31]. Contrary to our results, the study conducted by Jaberee SR et al. showed that perceived susceptibility was not a predictor of self-medication preventive behaviors [32].

Another study points out that the largest change was in perceived susceptibility, whereas the smallest changes were in perceived severity and perceived benefit [33]. The results are contradictory to those of other studies in which researchers used this model for education. The studies realized by Younis N et al. and Fathi M et al. showed that all HBM constructs play an important role in adopting preventive behaviors [34,35]. Regarding the mean level of perceived self-efficacy in another study, the main reason for using OTC medications, described by almost two-thirds of mothers, was to preserve their children’s lives in emergencies. The most commonly used medications were antipyretics, for reducing the child’s fever (91.9%) [36]. It was noted that caregivers with more children tend to self-medicate their children more compared to caregivers with their first child [37]. Another study reported that most of the caregivers agreed that it was important to give medicine to a child at home when he/she feels sick and medication given can prevent worsening of the disease [38].

Our study highlights the relationship between the level of satisfaction with the family doctor and the perceived threat of self-medication. In addition to primary care practices being the first line of support, information and support from professionals are highly valued [39]. Most people trust their family physician’s perspectives [40]. Another study showed that participants who were involved in decisions on how to manage their children’s illness by their GPs facilitated responsible use of antibiotics [41]. In studies conducted by Fathi et al. and Mohsen et al., it was found that physicians and health staff were effective in promoting preventive behaviors [35,42]. Physicians were also identified as the most important source of information, and educational programs had an important influence on the behavior of the parents concerning treating or preventing children’s diseases [43,44,45]. The study conducted by Biezen et al. shows that communication between GPs and parents is therefore a vital component in reducing antibiotic prescribing in general practice [46]. Low confidence in medical care systems increased the risk of antibiotic self-medication, and knowing prescription-only regulation for sales of antibiotics by community pharmacies was a protective factor against antibiotic self-medication in children [47]. The primary healthcare system, through family doctors, can effectively improve the continuity of care for patients with chronic diseases, and studies on diabetes reached this conclusion [48]. Other studies show that compliance with recommendations from health workers may also be correlated with confidence in vaccine efficacy because they can share personal knowledge about being immunized and motivate vaccine uptake efficacy [49].

Both traditional and recent health promotion interventions [50,51] have demonstrated that both public health campaigns and medical education provided in contexts such as doctor–patient interpersonal communication can have an impact on health-related behaviors. Regarding self-medication and parental education about preventing self-treating of their children, interventions targeting specific groups with personalized messages are sporadic. In this regard, our research has been able to show that providing a self-medication prevention message can bring about at least a change in the attitudes and opinions of a target group.

It is becoming increasingly evident that, in the future, healthcare institutions should start and engage in audience education activities [52] to tailor communication policies and healthcare delivery. Patients should be trained to acquire the skills to evaluate the structure of accurate and credible information to prevent the spread of contradictory medical information that could lead to disastrous consequences for the scientific authority of doctors and, consequently, public health. Providing medical education in doctor–patient encounters remains one of the most efficient and handy ways of delivering proper health-related behaviors.

Educating patients about responsible self-medication can bring several benefits. It helps avoid unnecessary visits to medical facilities for minor, seasonal, or repeated ailments in patients who have responded well to initial supervised therapy and do not have concurrent chronic conditions. It also enables early identification of non-responsive cases, leading to timely referral to emergency medical services in collaboration with the parent/patient. Moreover, it relieves doctors from evaluating ongoing conditions that can be effectively managed with repeatedly indicated therapies [2,53].

Based on both previous research and our findings, we can conclude that the Health Belief Model (HBM) is an effective theoretical and methodological tool for assessing self-medication practices more accurately.

### Strengths and Limitations

As far as we know, there is no other study conducted in Romania on the cognitive factors of parental self-medication using the Health Belief Model. Our current and previous studies raised awareness of the personal factors that lead to a higher prevalence of parental self-medication in Romania. Furthermore, our findings could contribute to better regulation of the practice of self-medication through public policies. Still, our research data remain limited and must be replicated in different sociodemographic groups and areas for a better understanding of the factors that might affect the use of self-medication in children among Romanian parents. Also, the conclusions of our study cannot be generalized to the Romanian population due to the non-representativeness of the sample.

## 5. Conclusions

In general, our respondents evaluate negatively the practices of self-medication, especially the treatment of their children without medical advice. However, their attitudes towards self-medication vary depending on their beliefs about administering certain medications, such as acetaminophen, which is frequently used when children have a fever.

As our data showed, a perceived level of satisfaction in relation to family physicians could be a significant way of controlling and avoiding the treatment of children without medical advice.

Furthermore, our findings suggest that the high level of awareness among respondents about the risks of self-medication can be attributed to the medical information campaigns conducted by various healthcare professionals. Therefore, future health education campaigns need to be targeted specifically, with messages that guide how to act in particular cases depending on the medication used and the child’s condition. In Romania, messages on self-medication from health professionals have generally been too general and thus, when combined with pharmacy medication procurement practices, can lead to a misunderstanding of how medications should be used.

## Figures and Tables

**Table 1:** Distribution of respondents according to socio-demographics (N = 210).

	Category	Unexposed *n* (%)	Exposed *n* (%)	Total *n* (%)	*p*-Value
Age	18–23 years	2 (2.0%)	0 (0%)	2 (1.0%)	<0.01 *
	24–29 years	5 (4.9%)	7 (6.5%)	12 (5.7%)	
	30–35 years	20 (19.6%)	41 (38.0%)	61 (29.0%)	
	36–41 years	35 (34.3%)	36 (33.3%)	71 (33.8%)	
	42–46 years	23 (22.5%)	22 (20.4%)	45 (21.4%)	
	46+	17 (16.7%)	2 (1.9%)	19 (9.0%)	
Sex	Females	94 (93.1%)	101 (93.5%)	195 (93.3%)	0.89 *
	Males	7 (6.9%)	7 (6.5%)	14 (6.7%)	
Residence	Urban	96 (94.1%)	78 (72.2%)	174 (82.9%)	0.01 *
	Rural	6 (5.9%)	30 (27.8%)	36 (17.1%)	
Education	University degree	84 (82.4%)	101 (93.5%)	185 (88.1%)	0.13 *
	Secondary school	18 (17.6%)	7 (6.5%)	25 (11.9%)	
Number of children	One Child	51 (50.0%)	58 (53.7%)	109 (51.9%)	0.34 *
	Two Children	45 (44.1%)	39 (36.1%)	84 (40.0%)	
	Three or more children	6 (5.9%)	11 (10.2%)	17 (8.1%)	

* Nominal data, *p*-value was calculated using chi-square test.

**Table 2:** Assessment of the medical system.

	Category	Mean ± SD	*p*-Value
Assessment of the GP’s activity	Unexposed	4.28 ± 1.07	0.48 *
	Exposed	4.18 ± 1.16	
Assessment of the health system	Unexposed	2.69 ± 1.08	0.89 *
	Exposed	2.44 ± 0.97	

* Numerical values, *p*-value was calculated using *t*-test.

**Table 3:** Variables measured according to the HBM model—differences between groups.

	Category	Mean ± SD	*p*-Value
It is hazardous to give my child medication without asking the doctor first.	Unexposed	3.91 ± 1.04	0.63 *
	Exposed	3.98 ± 1.08	
If I give medication to my child without medical advice, there can be consequences/complications for his/her health.	Unexposed	4.18 ± 0.99	0.28 *
	Exposed	4.32 ± 0.99	
I am capable of avoiding medicine self-administration without medical recommendation.	Unexposed	3.99 ± 1.14	0.34 *
	Exposed	3.83 ± 1.24	
If I follow the doctor’s advice, the child will heal/improve their health status faster.	Unexposed	4.45 ± 0.89	0.92 *
	Exposed	4.46 ± 0.87	
If I give a medicine (e.g., ibuprofen/paracetamol) without medical recommendation, the health status of the child still can improve.	Unexposed	3.82 ± 1.16	0.38 *
	Exposed	3.69 ± 1.12	
I do not have time to go to the doctor each time the child is sick.	Unexposed	2.37 ± 1.39	0.43 *
	Exposed	2.22 ± 1.36	

* Numerical values, *p*-value was calculated using *t*-test.

**Table 4:** Pearson correlation of perceived severity of self-medication and level of satisfaction with the GP.

		Perceived Severity of Self-Medication	Assessment of the GP
Perceived severity of self-medication	Correlation	1	0.258 *
	Sig. (2-tailed)		*p* < 0.01
	N	210	210

* Correlation is significant at the 0.01 level (2-tailed).

## Data Availability

Data are contained within the article and Supplementary Materials.

## Supplementary Materials

The following supporting information can be downloaded at: https://www.mdpi.com/article/10.3390/medicina59122093/s1, Supplementary Materials no. S1. Responses to the main topics of the questionnaire (N = 210).
