# Supplementary material for: Assessing the Effects of Medical Information on Parental Self-Medication Behaviors for Children’s Health: A Comparative Analysis
Source: Medicina (Kaunas). 2023 Nov 29;59(12):2093. doi: 10.3390/medicina59122093 (PMC10745013; doi:10.3390/medicina59122093)
Supplement: Supplementary file 1 [file medicina-59-02093-s001.zip › medicina-2673951-supplementary.pdf]

Supplementary material no. S1. Responses to the main topics of the questionnaire (N=210).

	Category	Unexposed n (%) N=102	Exposed n (% N=108	Total n (%) N=210
It is hazardous to give my child medication without asking the doctor first	Strongly disagree	2 (2.0%)	4 (3.7%)	6 (2.9%)
	Disagree	3 (2.9%)	3 (2.8%)	6 (2.9%)
	Neutral	38 (37.3%)	30 (27.8%)	68 (32.4%)
	Agree	18 (17.6%)	25 (23.1%)	43 (20.5%)
	Strongly agree	41 (40.2%)	46 (42.6%)	87 (41.4%)
If I give medication to my child without medical advice, there can be consequences/complications for his/her health	Strongly disagree	1 (1.0%)	3 (2.8%)	4 (1.9%)
	Disagree	4 (3.9%)	2 (1.9%)	6 (2.9%)
	Neutral	24 (23.5%)	17 (15.7%)	41 (19.5%)
	Agree	20 (19.6%)	21 (19.4%)	41 (19.5%)
	Strongly agree	53 (52.0%)	65 (60.2%)	118 (56.2%)
I am capable to avoid medicines self-administration without medical recommendation	Strongly disagree	6 (5.9%)	9 (8.3%)	15 (7.1%)
	Disagree	4 (3.9%)	6 (5.6%)	10 (4.8%)
	Neutral	18 (17.6%)	21 (19.4%)	39 (18.6%)
	Agree	31 (30.4%)	30 (27.8%)	61 (29.0%)
	Strongly agree	43 (42.2%)	42 (38.9%)	85 (40.5%)
If I follow the doctor's advice, the child will heal/improve their health status faster	Strongly disagree	2 (2.0%)	2 (1.9%)	4 (1.9%)
	Disagree	2 (2.0%)	3 (2.8%)	5 (2.4%)
	Neutral	9 (8.8%)	6 (5.6%)	15 (7.1%)
	Agree	24 (23.5%)	29 (26.9%)	53 (25.2%)
	Strongly agree	65 (63.7%)	68 (63.0%)	133 (63.3%)
If I give a medicine (e.g. ibuprofen/paracetamol) without medical recommendation the health status of the child still can improve	Strongly disagree	7 (6.9%)	7 (6.5%)	14 (6.7%)
	Disagree	4 (3.9%)	6 (5.6%)	10 (4.8%)
	Neutral	24 (23.5%)	29 (26.9%)	53 (25.2%)
	Agree	32 (31.4%)	38 (35.2%)	70 (33.3%)
	Strongly agree	35 (34.3%)	28 (25.9%)	63 (30.0%)
I do not have to ask the doctor when giving medicines like ibuprofen	Strongly disagree	16 (15.7%)	20 (18.5%)	36 (17.1%)
	Disagree	9 (8.8%)	22 (20.4%)	31 (14.8%)
	Neutral	35 (34.3%)	28 (25.9%)	63 (30.0%)
	Agree	17 (16.7%)	19 (17.6%)	36 (17.1%)
	Strongly agree	25 (24.5%)	19 (17.6%)	44 (21.0%)
If buying medicines without a medical prescription is legal, then medical advice is not needed	Strongly disagree	36 (35.3%)	67 (62.0%)	103 (49%)
	Disagree	24 (23.5%)	18 (16.7%)	42 (20.0%)
	Neutral	24 (23.5%)	14 (13.0%)	38 (18.1%)
	Agree	13 (12.7%)	3 (2.8%)	16 (7.6%)
	Strongly agree	5 (4.9%)	6 (5.6%)	11 (5.2%)
I do not have time to do to the doctor each time the child is sick	Strongly disagree	42 (41.2%)	48 (44.4%)	90 (42.9%)
	Disagree	14 (13.7%)	19 (17.6%)	33 (15.7%)
	Neutral	21 (20.6%)	20 (18.5%)	41 (19.5%)
	Agree	16 (15.7%)	11 (10.2%)	27 (12.9%)
	Strongly agree	9 (8.8%)	10 (9.3%)	19 (9.0%)
